# Iliopsoas hematoma secondary to small needle scalpel for the treatment of nonspecific low back pain: A case report

**DOI:** 10.1097/MD.0000000000031975

**Published:** 2022-11-18

**Authors:** Wu Zeng, XiaoMing Zhou, JunFeng Zhu, Jun Li, YongYong Weng

**Affiliations:** a Suichang County People’s Hospital, SuiChang, ZheJiang, China.

**Keywords:** acupuncture, case report, iliopsoas hematoma, low back pain

## Abstract

Nonspecific low back pain (NSLBP) is one of the most common symptoms which can happen all ages and it accounts for the majority of low back pain (LBP). Current clinical studies have shown that rest, physical therapy, acupuncture (including small needle scalpel) and non-steroidal antiinflammatory drugs are effective treatments for NSLBP. However, the safety of small needle scalpel for treatment of NSLBP was reported rarely.

**Patient concerns:**

A 63-year-old female patient was referred to the emergency department for right lower back pain, right lower quadrant ache, weakness of flexion right hip joints and worsening pain with walking after the treatment of small needle scalpel, which was performed by a rural doctor; the symptoms had been lasting for 9 hours.

**Diagnosis:**

She was diagnosed with traumatic iliopsoas hematoma because she experienced increased back pain after accepting small needle scalpel. Clopidogrel was stopped and the patient did not received a blood transfusion and just monitored Blood routine examination, liver and function, coagulation function after admission.

**Interventions:**

She had rest in bed absolutely for 3 days after admission. On the fourth day, she restarted taking Clopidogrel 75 mg every day and has gradually increased time for ambulation. She was discharged home and was ambulating with the help of a walking frame on day 7 and her follow-up abdominal CT scan on day 11 revealed reduced slightly hematoma. She was treated with rest, and showed an gradual recovery in approximately 3 weeks.

**Outcomes:**

At day 85, the patient’s LBP symptoms had completely disappeared and the result of liver function, renal function, coagulation function, blood routine was normal.

**Conclusion:**

Small needle scalpel is a form of acupuncture. In China, small needle scalpel therapy has been used to treat various kinds of chronic pain. Anticoagulation therapy is a risk for bleeding, and patients who used Clopidogrel prepare to adopting small needle scalpel needs to be very cautious.

## 1. Introduction

Between 80% and 85% of the population suffer low back pain (LBP) at some point in their lifetime.^[1]^ Nonspecific low back pain (NSLBP) is one of the most common symptoms which can happen at all ages and it accounts for approximately 85% of LBP.^[2,3]^ It is most commonly found in middle-aged females.^[4]^ What’s more, 1 year recurrence rate was between 24% and 80%.^[5]^ It is also one of major contributor to the increasing global disease burden and is associated with substantial downstream economic losses and reduced quality of life.^[6]^ The most frequently used treatment options involves rest, physical therapy, acupuncture (including Small needle scalpel) and non-steroidal antiinflammatory drugs.^[7–9]^ In complementary and alternative medicine, acupuncture have been increasingly used in patients with LBP and it might more effective in improving pain and function.^[10–12]^ The National Institutes for Health and The American Academy of Family Physicians recognize acupuncture as an important option for LBP.^[7–9]^ Small needle scalpel is a form of acupuncture. It was introduced in China from 1976. In China, small needle scalpel therapy has been used to treat various kinds of chronic pain including nonspecific low back or neck pain, shoulder pain, chronic headache/migraine or osteoarthritis.^[13–16]^ It is a technique that combines both acupuncture and microinvasive surgery.^[17]^

Iliopsoas hematoma is an uncommon complication and it has numerous causes including antithrombotic therapy, damage due to exercise or injury, and other unidentified factors.^[18–20]^ Among them the most common of which is spontaneous iliopsoas hematoma and Most clinical studies of iliopsoas hematoma were derived from case reports.^[15,16,21,22]^ For the moment, no reports have suggested a relationship between small needle scalpel and IPH, and this is the first report to demonstrate that IPH may be a complication of small needle scalpel.

## 2. Case presentation

**Figure 1.**
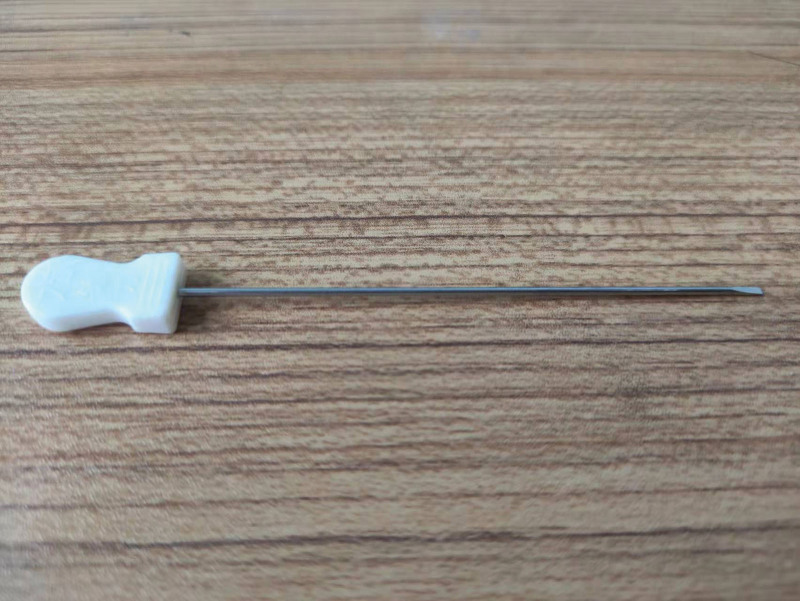
Photograph of the small needle scalpel. The shape of the small needle scalpel looks like a needle for acupuncture with a sharp bladed tip and 0.4 to 1.2 mm in diameter.

A 63-year-old female patient was referred to the emergency department for right lower back pain, right lower quadrant ache, weakness of flexion of right hip joints and worsening pain with walking. The patient complained of NSLBP since approximately last month and the symptoms appeared to get worse after the treatment of a small needle scalpel (Fig. 1), which was performed by a rural doctor; the symptoms had been lasting for 9 hours. Her height was 148 cm, and her body weight was 50.6 kg. No femoral nerve paralysis was observed. She was diagnosed with traumatic iliopsoas hematoma because she experienced increased back pain after accepting a small needle scalpel. She had not only a history of hypertension over 7 years ago, but had a history of diabetes for 2 years. Coronary artery bypass grafting was performed after coronary heart disease was diagnosed 7 years ago and she started taking Clopidogrel 75mg every day for antiplatelet therapy. Other long-term medications before admission were isosorbide mononitrate, rosuvastatin Calcium, perindopril Tert-Butylamine, extended-Release metoprolol, and acarbose.

Admission physical examination revealed the patient to be afebrile (36.5°), normal pulse of 100/min, a respiratory rate of 20/min, and blood pressure of 112/69 mm Hg. The abdominal tenderness point was positioned in the right lower abdomen. The groin felt pain when the right leg with flexion of knees and hip joints was straightened in supine position. The initial laboratory data revealed slightly lower hemoglobin (Hb 112g/L; normal references:115~150 g/L) and the coagulation function test of study group was normal and liver and renal function test was normal. Blood glucose was 11.84 mmol/L (normal references: 4.10~5.90 mmol/L) and it significantly higher than normal reference. Glycosylated Hb A1c was 6.9% (normal references: 4.00%~6.00%). Her systemic examination and blood workups were otherwise unremarkable.

**Figure 2.**
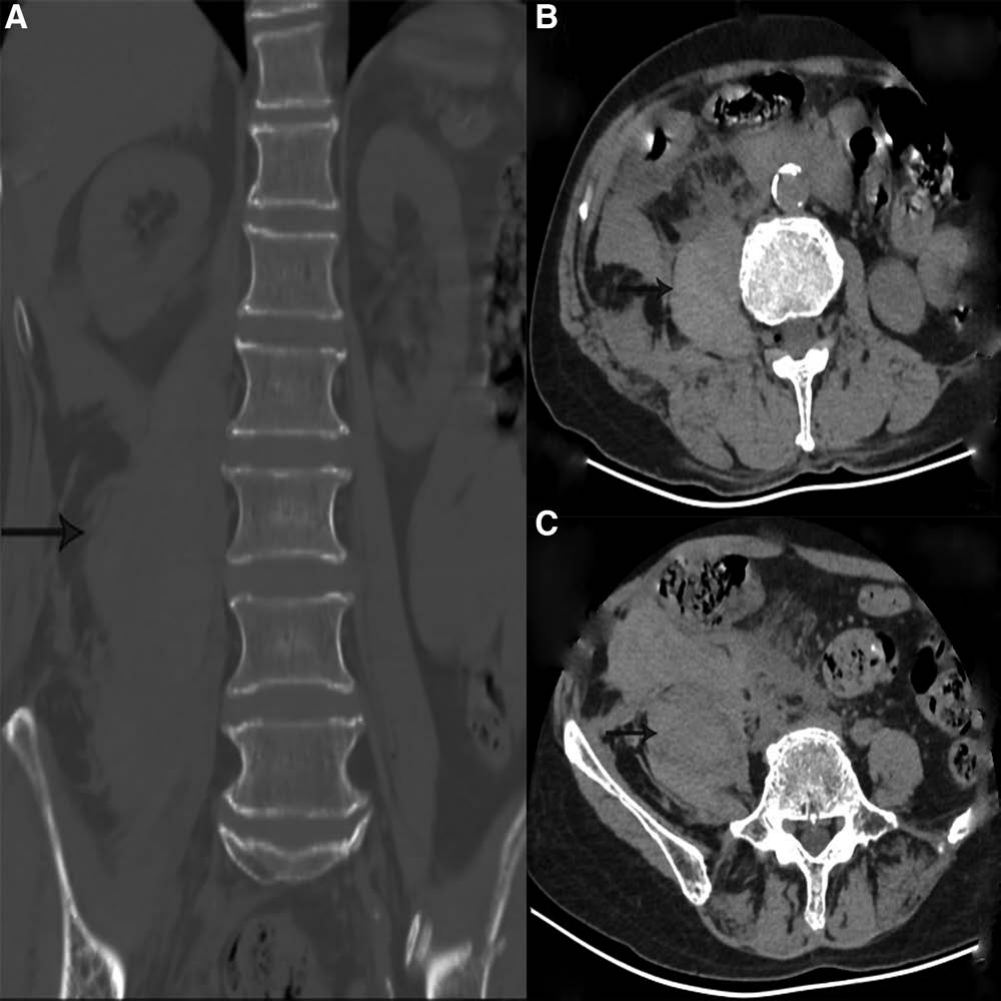
Plain CT image of the lumbar intervertebral disc obtained about 7 h after the treatment of small needle scalpel. (a) Coronal view; (b) horizontal view at the L2 level; (c) horizontal view at the L5 level. CT = computed tomography.

**Figure 3.**
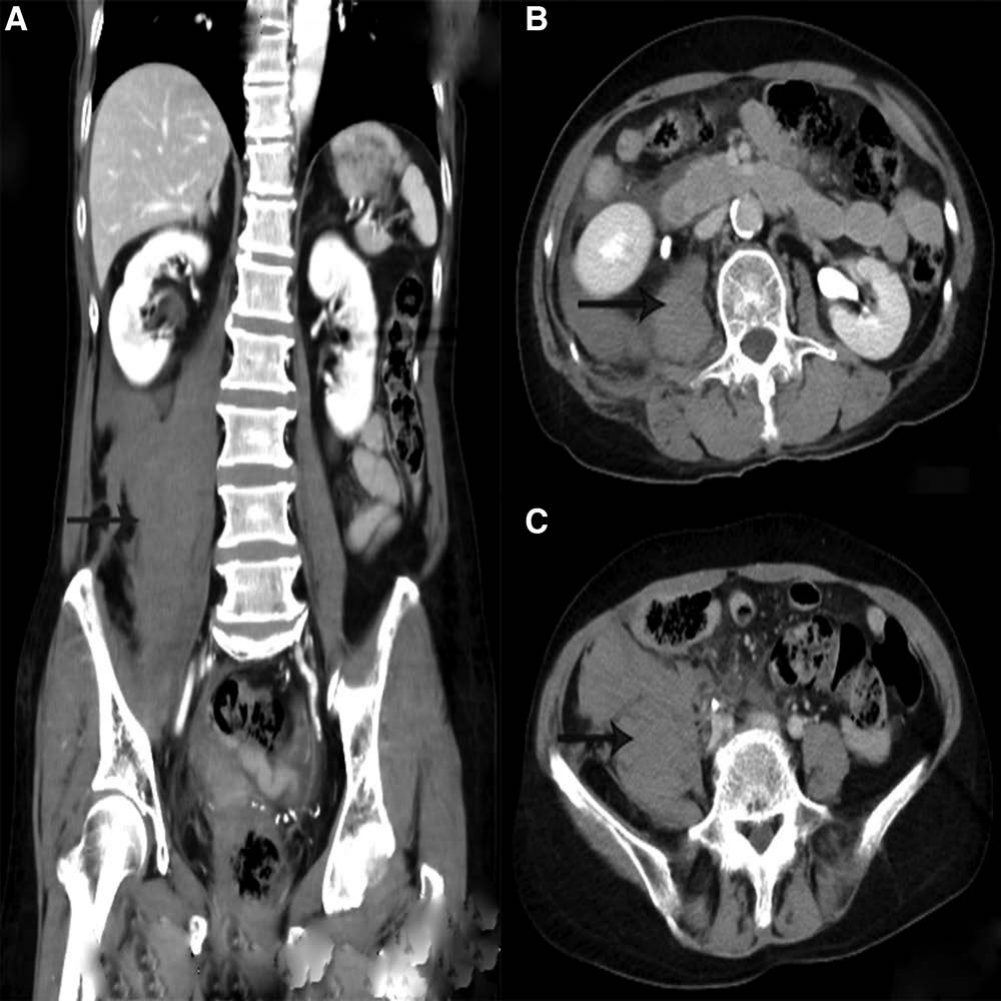
Contrast-enhanced CT image of the abdomen obtained about 9 h after the treatment of small needle scalpel. (a) Coronal view; (b) horizontal view at the L2 level; (c) horizontal view at the L5 level. CT = computed tomography.

At 17:18, computed tomography (CT) of the lumbar intervertebral disc had detect some abnormal findings, such as swelling of the iliopsoas muscle, the right retroperitoneal hematoma, iliac hematoma and hemoperitoneum (Fig. 2a–c). At 18:20, during dynamic abdominal contrast-enhanced CT, a hematoma was detected in the right iliopsoas muscle at the L2 and L5 levels (Fig. 3a–c). Clopidogrel was stopped and the patient did not receive a blood transfusion and just monitored Blood routine examination, liver and function, coagulation function after admission. Contrast medium was selectively injected into the aorta, renal arteries, inferior mesenteric artery, and internal and external iliac arteries, but no contrast media pooling was detected.

She had rested in bed absolutely for 3 days after admission. She continued to have right lower back pain, right lower quadrant ache, and weakness of flexion of right hip joints but part of her blood workups was abnormal on day 1. Blood routine examination showed red blood cell (3.50 × 1012/L; normal references: 3.80~5.10 × 1012/L), Hb (Hb 93g/L; normal references: 115~150 g/L) and Ht decreased significantly (Ht 0.283 L/L; normal references: 0.350~0.450 L/L).

Although retroperitoneal bleeding is a well-recognized complication of clopidogrel use, use of antiplatelet drugs has shown consistent benefit in the prevention of ischemic stroke, myocardial infarction and vascular death in patients, and what’s more important is that clopidogrel has a half-life of 7 to 8 hours.^[23,24]^ Accordingly, on day 4, she restarted taking Clopidogrel 75 mg every day and gradually increased time for ambulation.

**Figure 4.**
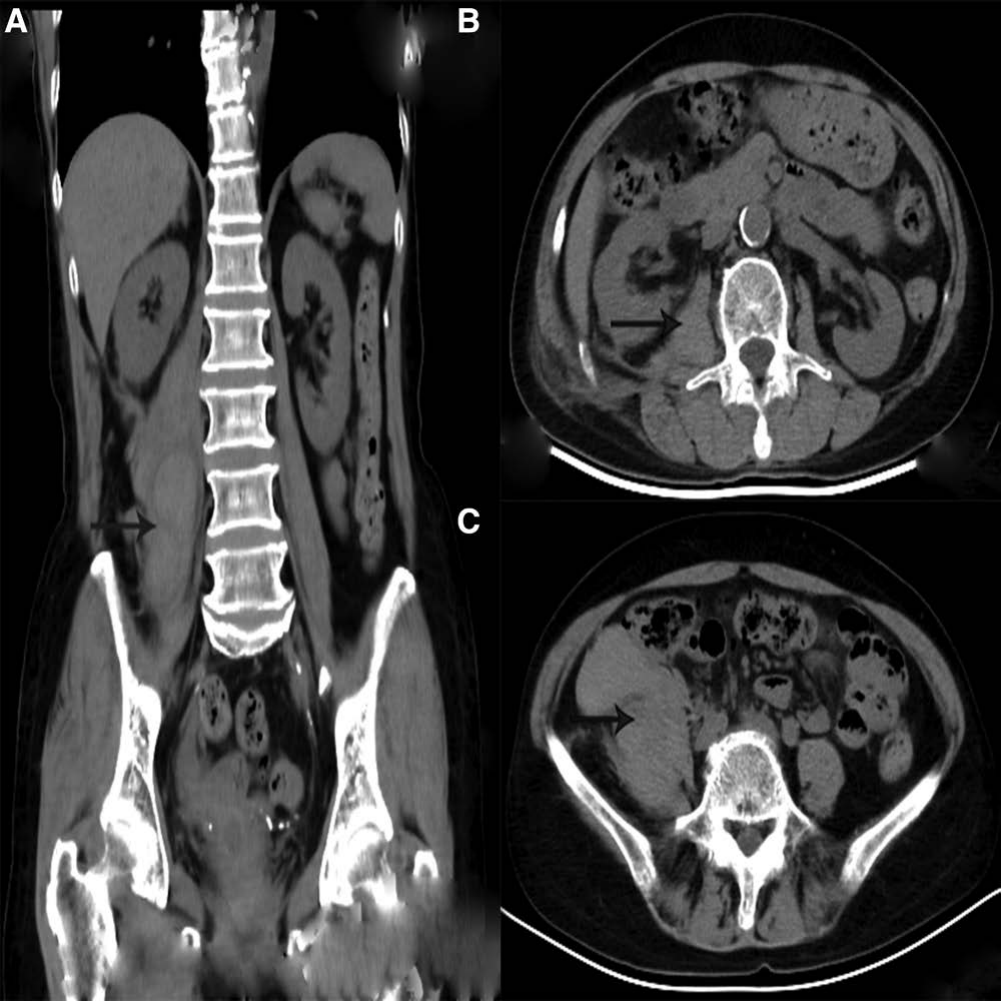
Plain CT image of the abdomen obtained 6 d after the treatment of small needle scalpel. (a) Coronal view; (b) horizontal view at the L2 level; (c) horizontal view at the L5 level. CT = computed tomography.

**Figure 5.**
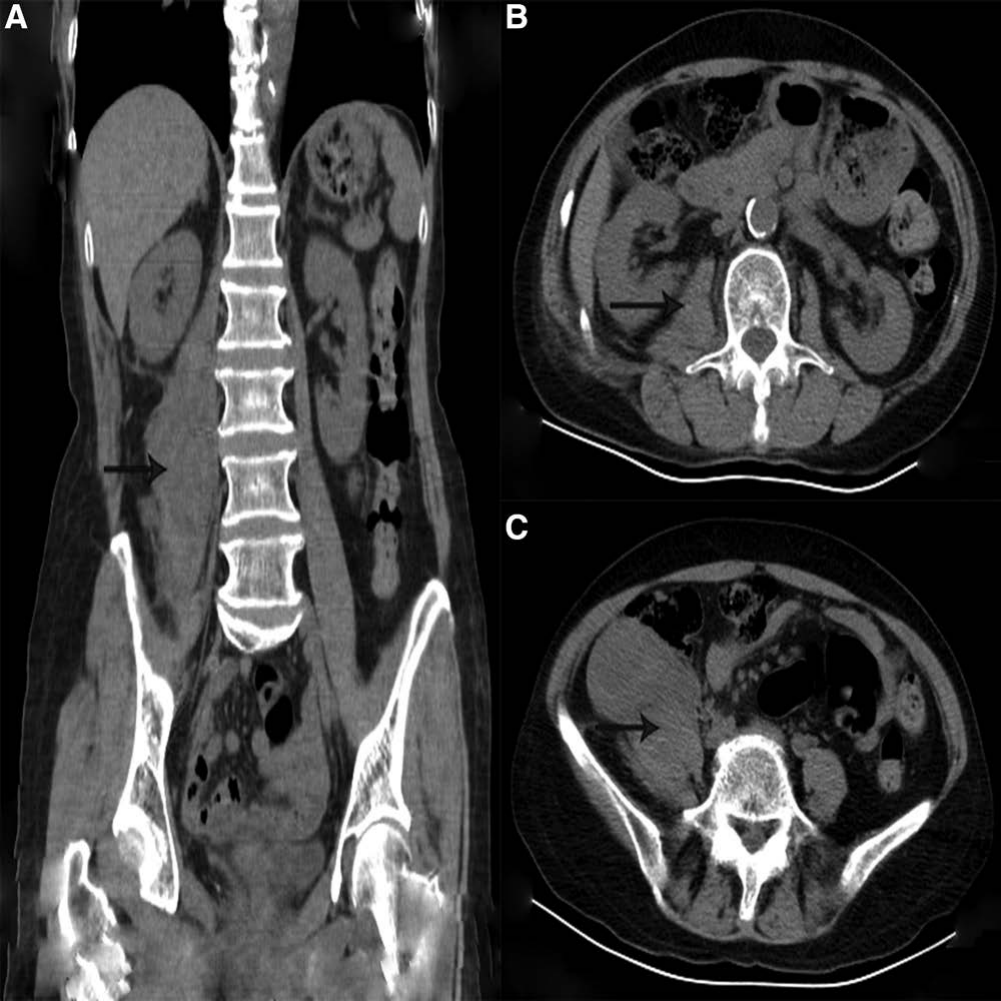
Plain CT image of the abdomen obtained 11 d after the treatment of small needle scalpel. (a) Coronal view; (b) horizontal view at the L2 level; (c) horizontal view at the L5 level.

She continued to have right lower back pain, right lower quadrant ache, weakness of flexion right hip joints and blood tests detected that the patient’s Hb level had fallen to 85 g/L on day 6. Moreover, red blood cell (3.19 × 1012/L) and Ht decreased significantly (Ht 0.260 L/L). CT was repeated and the iliopsoas muscle was similar compared with its size on the CT scan in emergency department (Fig. 4a–c). On day 7, during her hospitalizations, she did not receive a blood transfusion. She was discharged home and was ambulating with the help of a walking frame on day 7 and her follow-up abdominal CT scan on day 11 revealed reduced slightly hematoma (Fig. 5a–c). The patient described in the present paper was treated with rest and showed a gradual recovery in approximately 3 weeks. On day 85, her LBP symptoms had completely disappeared and the result of liver function, renal function, coagulation function, and blood routine was normal.

## 3. Discussion

In this case, initially diagnosed with NSLBP the patient reported here received the small needle scalpel treatment to relieve pain while being treated by a rural medical doctor. We hypothesize that small needle scalpel for the treatment of NSLBP can cause iliopsoas muscle hematoma, and the hematoma could be caused by direct blood vessel damage during the small needle scalpel operation. It’s based in its most powerful part on the fact that the bleeding occurred near the surgical site and the hematoma was confirmed by CT examination in emergency departments. What’s more, first clinical symptoms occur several hours after the precipitating event of such a hematoma. This may be the explanation of why our patient reported her first complaints several hours after treatment with small needle scalpel. Although this patient had a history of using Clopidogrel, the coagulation function test results were normal in emergency departments. Commonly reported risk factors for IPH to include trauma, anticoagulation therapy, and hemodialysis.^[18–20]^ Iliopsoas muscle hematomas caused by trauma are reported, but no reports described iliopsoas muscle hematoma after small needle scalpel for the treatment of NSLBP has been found.

It was surveyed in 2016, LBP was the leading cause of disability in the whole year globally, affecting 57.6 million people.^[25]^ Among older adults chronic LBP is associated with functional decline, falls, and increased mortality.^[26]^ In the United Kingdom, it was found that the national economic burden of LBP was similar to that of high-cost diseases such as cardiovascular disease, cancer and autoimmune disease.^[27,28]^ Small needle scalpel is a form of acupuncture. Acupuncture has shown favorable effects in the clinical treatment of chronic pain including nonspecific low back or neck pain, shoulder pain, chronic headache/migraine or osteoarthritis.^[29,30]^ Small needle-knife therapy is a technique that combines both acupuncture and microinvasive surgery. Other names include acupotomy, needle scalpel, mini-scalpel acupuncture and mini-needle-knife, and mini-scalpel needle.^[31]^

CT is a fast, highly sensitive, and commonly used imaging modality to diagnose spontaneous iliopsoas hematoma. Iliopsoas hematoma is a rare and life-threatening complication of bleeding disorders that occurs in patients with clotting disorders and also in association with anticoagulant drug treatments.^[32,33]^ The symptoms of iliopsoas muscle hematoma include inguinal pain, flexion of the hip, femoral nerve palsy, mass formation in the iliac fossa, bleeding-related anemia, and constipation.^[34]^ In the present case, the patient’s main symptoms were right lower back pain, right lower quadrant ache, weakness of flexion right hip joints. Tenderness or swelling of the inguinal region was observed without femoral nerve palsy.

In conclusion, the reason for the iliopsoas hematoma of the present case was damage caused by small needle scalpel. Although the benefits of small needle-scalpel therapy have been confirmed, such as reducing pain, shorter expenditure, shorter period of therapy and better recovery of function, the complications have not so far paid too much attention. Therefore, the possibility of IPH as a complication of small needle scalpel for the Treatment of NSLBP should be considered before and during the administration of anticoagulation therapy.

## 4. Limitations

Although coagulation is normal in emergency departments, the impact of iliopsoas hematoma secondary to small needle scalpel for the current case cannot be separated from the possibility of the effects of clopidogrel on bleeding risk.

## 5. Conclusion

Small needle scalpel is a form of acupuncture. In China, small-needle scalpel therapy has been used to treat various kinds of chronic pain. Anticoagulation therapy is a risk for bleeding and patients who used Clopidogrel preparing to adopt small needle scalpel need to be very cautious.

## Authors contribution

**Data curation:** YongYong Weng.

**Investigation:** Jun li.

**Writing – original draft:** Wu Zeng, XiaoMing Zhou.

**Writing – review & editing:** JunFeng Zhu.
